# Evaluating artificial intelligence large language models in dental education: a cross-sectional survey on usage, perceptions, and integration at a U.S. dental school

**DOI:** 10.3389/fdgth.2026.1786363

**Published:** 2026-06-08

**Authors:** Celine Sheng, Camie McFarland, Nikola Angelov, Sridhar V. K. Eswaran, Richard Halpin, Jennifer Chang

**Affiliations:** 1 Department of Periodontics and Dental Hygiene, UTHealth School of Dentistry at Houston, Houston, TX, United States; 2 Pre-Doctoral Program, UTHealth School of Dentistry at Houston, Houston, TX, United States; 3 Office of Technology and Services and Informatics, UTHealth School of Dentistry at Houston, Houston, TX, United States

**Keywords:** artificial intelligence, attitude of health personnel, dental, education, educational technology

## Abstract

**Introduction:**

The adoption of artificial intelligence (AI) in higher education presents opportunities and challenges for dental education. This study explores the use of Large Language Model (LLM) based AI tools, including ChatGPT and Grammarly AI, among faculty and students at the UTHealth School of Dentistry in Houston (UTSDH). This research assessed usage patterns, perceived benefits and concerns, and AI training demand.

**Methods:**

A piloted, cross-sectional survey was administered via email. The survey included Likert-scale, multiple-choice, and open-ended questions. Respondents provided demographics and rated their LLM-based AI tools for use and perceptions in educational, clinical, and research contexts. Data was analyzed using Kruskal–Wallis and Tukey–Kramer–Nemenyi tests. Qualitative responses were analyzed thematically.

**Results:**

Among 243 respondents, 66% of faculty and 73% of students reported using LLM-based AI tools, primarily for writing and educational tasks. Students were more likely to perceive LLM-based AI tools as beneficial (*p* < 0.01), while faculty showed stronger demand for AI training (*p* < 0.05). Gender differences were significant, with males were more supportive of AI in research tasks (*p* < 0.05). User experience ratings differed, with students rating ChatGPT more favorably across all categories.

**Discussion:**

LLM-based AI tools (e.g., ChatGPT and Grammarly AI) are becoming increasingly relevant in dental education, particularly in academic writing and concept learning. While students are leading early adoption, faculty expressed a strong need for structured AI training, highlighting fertile ground for development programs. Clinical and research applications remain underdeveloped but promising. Addressing these gaps through tailored education, ethical guidelines, and institutional support will be essential for optimizing AI's potential across dental education and practice.

## 1 Introduction

Despite AI's transformative impact on various healthcare domains, its role in dental education remains insufficiently studied. Large language models (LLMs), such as ChatGPT (OpenAI, San Francisco, California, U.S.) and Grammarly AI (Grammarly Inc., San Francisco, California, U.S.), have demonstrated utility in improving administrative and educational efficiency, offering potential benefits for dental students and faculty (1). However, questions persist regarding ethical considerations, the risk of overreliance, and the broader integration of these tools into both educational and clinical practice. While previous studies have emphasized LLM-based AI's promise in diagnostics and treatment planning, there has been limited focus on its adoption in dentistry-specific educational contexts (1, 2).

LLMs are a subset of artificial intelligence that specializes in understanding and generating human-like text based on vast amounts of data. They are built using advanced machine learning techniques and are broadly categorized into two types: those that are primarily used for natural language understanding (NLU) and those that focus on natural language generation (NLG). In our survey, we concentrate on the NLG type of LLM, as exemplified by ChatGPT and Grammarly AI, which are designed to generate coherent and contextually relevant responses, making it particularly useful for educational applications (3).

This study is the first to comprehensively survey both the perceptions and real-world usage of LLM-based AI tools among faculty, residents, dental and hygiene students within a U.S. dental school. By including these diverse groups, it provides a nuanced view of current AI applications and perspectives on future integration (4, 5). Previous research in dental education has highlighted gaps in AI confidence between faculty and students, insufficient standardized training, and limited familiarity with advanced AI concepts, revealing a broader need for curriculum development (5, 6). However, much of the existing literature has focused mainly on general awareness rather than practical adoption. The present investigation moves beyond these limitations by examining actual use of AI in the educational environment, mapping the extent of integration, and offering insights to guide ethical and effective curriculum evolution (7, 8). This study had three main objectives: (1) to investigate and compare the utilization of LLM-based AI applications among dental faculty, residents, and dental and dental hygiene students at UTSDH; (2) to analyze differences in attitudes and perspectives toward LLM-based AI applications between faculty and students; and (3) to explore the potential role of LLM-based AI applications in dental education and research.

## 2 Materials and methods

An online survey consisting of structured questionnaires and three open-ended question was developed by the authors in accordance with established survey design standards and responsiveness guidelines, as well as prior AI-in-dental-education survey methodologies to enhance validity and comparability (9–11). The instrument was pilot tested among a small group of faculty and students at the University of Texas School of Dentistry at Houston (UTSDH) to identify and resolve potential ambiguities prior to distribution. The full survey is provided in the Supplementary Material.

The final survey was administered via institutional email lists to full-time and part-time faculty, residents, dental students, and dental hygiene students affiliated with UTSDH. Of 864 invited participants (220 faculty, 422 dental students, 113 residents, and 56 dental hygiene students), 243 completed the survey, yielding a response rate of 28.1%. Data were collected anonymously using QuestionPro (QuestionPro Inc., Austin, TX), a secure web-based platform enabling controlled distribution and export analysis.

Survey items were organized into four domains: (1) patterns of large language model (LLM)-based AI use, (2) perceived benefits and risks, (3) acceptability of AI integration in educational, clinical, and research contexts, and (4) perceived training needs. The study was approved by the Institutional Review Board of UTHealth School of Dentistry at Houston (HSC-DB-23-0814), and informed consent was obtained from all participants prior to participation.

### 2.1 Quantitative analysis

Data were analyzed using R. Ordinal variables were summarized using medians and interquartile ranges (IQR). Differences between groups were assessed using the Kruskal–Wallis rank-sum test for comparisons involving three or more independent groups (e.g., role or department), followed by Tukey–Kramer–Nemenyi *post-hoc* pairwise comparisons when the overall test indicated significance (9). For two-group comparisons (e.g., gender), the Mann–Whitney *U* test was used. Categorical variables were reported as frequencies and percentages. A two-sided *p*-value <0.05 was considered statistically significant. All analyses were conducted within an exploratory framework.

### 2.2 Qualitative analysis

Open-ended responses were analyzed using inductive thematic analysis. A trained member of the research team conducted open coding through iterative review of the data, followed by grouping codes into categories and development of overarching themes using a constant comparative approach. Representative quotations were selected to illustrate themes. Given the exploratory scope of the qualitative component, coding was performed by a single analyst.

## 3 Results

A total of 243 out of 864 invited individuals completed the survey, yielding an overall response rate of approximately 28.1%. Participants were drawn from various academic roles, with faculty comprising 48.9% (*n* = 119) and students representing 51.0% (*n* = 124) of the respondents. The student cohort included dental students (*n* = 81), residents (*n* = 36), and hygiene students (*n* = 7).

For certain tables and figures, dental students, residents, and hygiene students were combined into a single “student” category to facilitate comparisons with faculty. In instances where these student subgroups were not combined, the specific populations included in the analysis are detailed within the respective table and figure legends.

Demographic data (Table 1) revealed a diverse participant pool. Among the 224 respondents who provided gender information, 137 identified as female, 84 as male, and 2 as non-binary/third gender, with 1 preferring not to state. 19 respondents opted not to answer the gender question. Age distribution varied by academic role; faculty respondents were primarily aged between 27 and 79 years (mean: 53 years), while students were predominantly between 22 and 43 years old (mean: 32 years). It is important to note that response totals for individual demographic questions varied due to their optional nature, a strategy employed to enhance overall survey completion rates.

**Table 1:** Demographic data.

Demographic Characteristics	Group		Number	Total number
Academic role	Faculty		119	243
	Student	Dental student	81	
		Hygiene student	7	
		Resident	36	
Gender	Male		84	224
	Female		137	
	Non-binary		2	
	Prefer not to say		1	
	Did not answer this question		19	
Age	Faculty (mean/range)		53/27–79	
	Student (mean/range)		32/22–43	

### 3.1 Usage and frequency

The survey included a dichotomous question indicating that most faculty and students had used or were currently using an LLM-based AI tool for dental education. Frequency of use was compared between faculty and dental students across different LLM-based AI platforms among respondents who answered “yes” to having used AI tools for dental education. LLM-based AI platforms that were in the survey included Bard, now known as Google Gemini (Gemini Trust Company, LLC, New York City, New York, U.S), Bing Chat, now known as Microsoft Copilot (Microsoft, Redmond, Washington, U.S.), ChatGPT, Claude (Anthropic, San Francisco, California, U.S.), Grammarly AI (Grammarly Inc. San Francisco, California U.S.), Jasper (Jasper AI Inc., Austin, Texas, U.S), and Perplexity (Perplexity AI, Inc., San Francisco, California, U.S).

**Figure 1:**
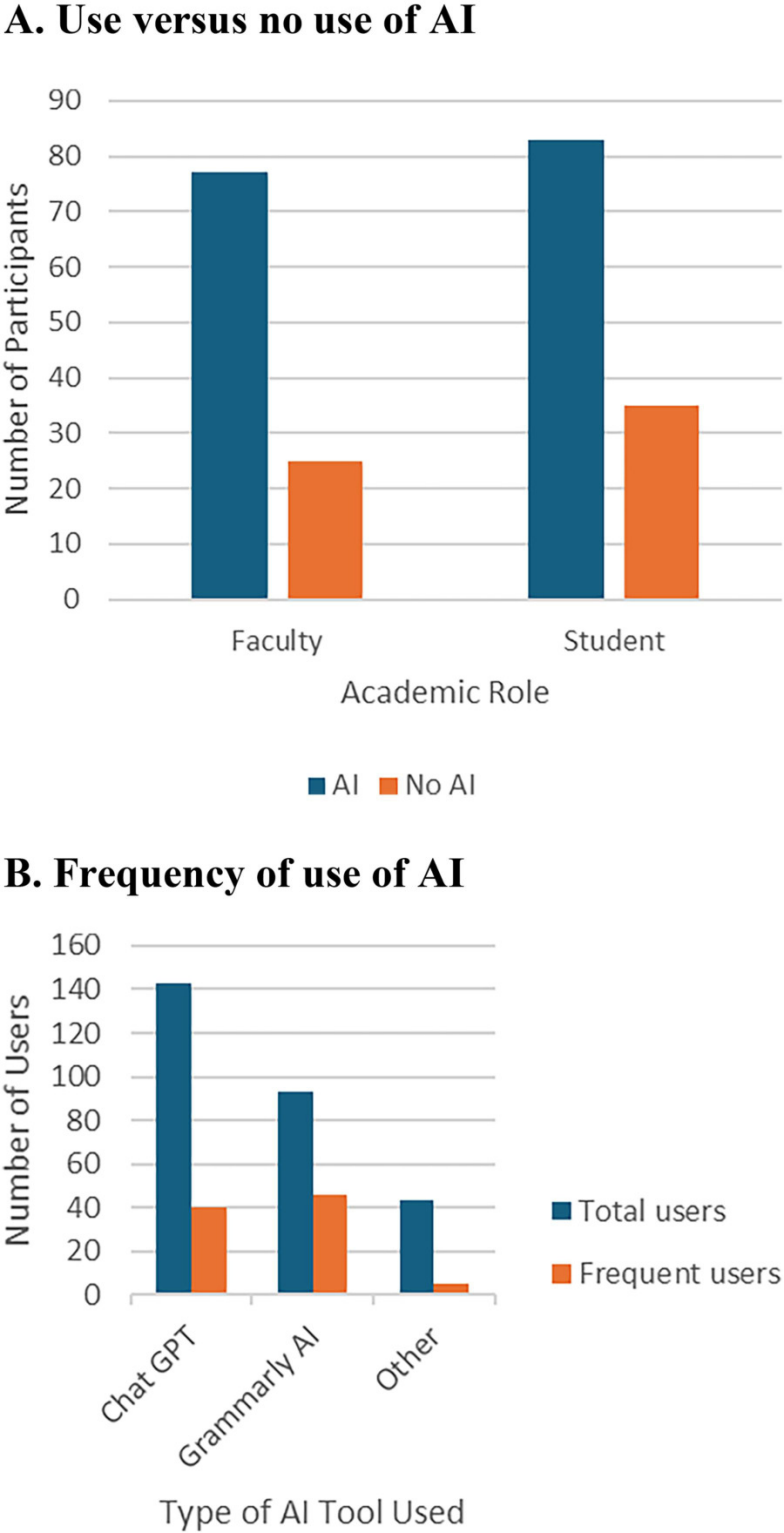
Use of AI. **(A)** Use vs. no use of AI. Out of the 119 faculty members who participated in this survey, 102 reported whether they used AI or not at work. Of which, 77 use AI at work and 25 do not. Out of 124 students (dental students, hygiene students, and residents) who participated in this survey, 118 reported whether they use AI or not at work. Of which, 83 used AI at work and 35 did not. **(B)** Frequency of use of AI. Total users (any use, combination of rarely, sometimes, often, or always): there were 143 users of ChatGPT, 93 users of Grammarly AI, and 43 users of other tools. Frequent users (often and always): there were 40 frequent users of ChatGPT, 46 users of Grammarly AI, and 5 users of other AI tools. Bard, Bing Chat, Claude, Jasper, Perplexity, and other tools are pooled into the other category due to low usage.

Regarding the overall usage of AI tools among the respondents, ChatGPT was the most widely used, with 143 individuals reporting any level of use (ranging from rarely to always). Grammarly AI followed with 93 users, and a combined category of “other tools” accounted for 43 users. When examining the frequency of use, specifically those who reported using tools “often” or “always,” Grammarly AI showed a slightly higher number of frequent users at 46. ChatGPT had 40 frequent users, while the “other AI tools” category had 5 frequent users. It's important to note that the “other tools” category consolidates platforms such as Bard, Bing Chat, Claude, Jasper, and Perplexity due to their individually lower usage rates (Figure 1).

Both ChatGPT and Grammarly AI demonstrate distinct yet complementary roles in educational, administrative, and research settings, while their application in clinical tasks remains notably limited (Supplementary Table S1). In educational and administrative contexts, ChatGPT is primarily utilized by students for understanding complex medical and dental concepts (20.65%), summarizing articles (12.90%), and assisting with presentation design (11.61%). Faculty, on the other hand, leverage ChatGPT for general communication (20.29%) and professional writing (15.94%). Grammarly AI is predominantly favored by both students and faculty for refining various forms of written communication; for students, this includes formal writing like reports and CVs (25.33%), essay responses (24.00%), and general emails/texts (21.33%). Faculty similarly rely on Grammarly AI for writing communication (36.05%) and professional documents (26.74%). For clinical tasks, a significant majority of users for both tools report no engagement, with 68.64% for ChatGPT and 71.79% for Grammarly AI, reflecting a cautious approach to AI in direct patient care. When used clinically, ChatGPT is occasionally consulted for pharmacological information (9.32%) and procedural instructions (5.93%), while Grammarly AI sees minimal application in patient communication (7.69%) and progress notes (7.69%). In the research domain, ChatGPT is frequently employed for summarizing literature (21.38%), finding references (13.79%), and drafting manuscripts (12.41%). Grammarly AI's primary contribution here is in the meticulous writing and refinement of articles and manuscripts (28.30%), alongside supporting grant applications (5.66%) and literature summarization (15.09%). This overall pattern highlights a shared confidence in AI for enhancing learning, communication, and research support, but a clear reservation regarding its direct involvement in critical clinical decision-making.

### 3.2 Perceptions of LLM-based AI tools in dental education

When participants were asked to rate whether the use of AI in dental care is ethical, cheating, beneficial, or efficient, 99–103 faculty members and 78 students responded to the items. Overall, both faculty and students reported generally positive or neutral perceptions, agreeing that AI use is ethical, beneficial, and efficient, while largely disagreeing that it constitutes cheating.

Group differences were assessed using the Kruskal–Wallis rank-sum test with Tukey–Kramer–Nemenyi *post-hoc* pairwise comparisons. No significant differences were observed for perceptions of ethicality, cheating, or efficiency. However, perceived benefit was the only domain showing a statistically significant difference between groups (*p* < 0.05), with a higher proportion of students agreeing or strongly agreeing that AI is beneficial compared with faculty.

Overall, while both groups demonstrated favorable views of AI integration in dental care, students expressed significantly stronger perceptions of its benefits than faculty (*p* < 0.05).

### 3.3 Acceptability of AI use by students and faculty

Across educational, clinical, and research domains, both faculty and students generally supported the use of LLM-based AI tools, with most responses falling in the “agree” or “strongly agree” categories (Table 2). Support was highest for research applications, where 59.2% of faculty and 58.5% of students agreed that students should use AI, and 61.2% and 57.6%, respectively, agreed for faculty use, with an additional 15.5%–22.3% strongly agreeing. Educational use was also highly endorsed (e.g., student use: faculty 52.4% agree, 11.7% strongly agree; students 48.3% agree, 17.8% strongly agree), whereas clinical use showed comparatively lower but still favorable support, with higher neutral responses (20%–24%). Overall, differences between faculty and students were modest, and results were descriptive.

**Table 2:** Should AI be used.

Task type	Survey Questions	Strongly disagree *n* (%)		Disagree *n* (%)		Neutral *n* (%)		Agree *n* (%)		Strongly agree *n* (%)	
Educational	Should students be allowed to use AI for educational tasks?	F	3	F	17	F	17	F	54	F	12
			2.9%		16.5%		16.5%		52.4%		11.7%
		S	5	S	15	S	20	S	57	S	21
			4.2%		12.7%		16.9%		48.3%		17.8%
	Should faculty be allowed to use AI for educational tasks?	F	0	F	1	F	20	F	61	F	21
			0.0%		1.0%		19.4%		59.2%		20.4%
		S	3	S	7	S	17	S	71	S	20
			2.5%		5.9%		14.4%		16.2%		16.9%
Clinical	Should students be allowed to use AI for educational tasks?	F	5	F	15	F	25	F	46	F	12
			4.9%		14.6%		24.3%		44.7%		11.7%
		S	3	S	17	S	28	S	56	S	14
			2.5%		14.4%		23.7%		47.5%		11.9%
	Should faculty be allowed to use AI for educational tasks?	F	2	F	9	F	21	F	53	F	18
			1.9%		8.7%		20.4%		51.5%		17.5%
		S	5	S	12	S	25	S	60	S	16
			4.2%		10.2%		21.2%		50.8%		13.6%
Research	Should students be allowed to use AI for educational tasks?	F	2	F	4	F	20	F	61	F	16
			1.9%		3.9%		19.4%		59.2%		15.5%
		S	2	S	9	S	16	S	69	S	22
			1.7%		7.6%		13.6%		58.5%		18.6%
	Should faculty be allowed to use AI for educational tasks?	F	0	F	0	F	17	F	63	F	23
			0.0%		0.0%		16.5%		61.2%		22.3%
		S	2	S	6	S	16	S	68	S	26
			1.7%		5.1%		13.6%		57.6%		22.0%

221 total responses from both questions are reported as respondent totals within each respondent group on a 5-point Likert scale. The student group combines dental students, dental hygiene students, and residents (*n* = 118); Faculty (*n* = 103). The percentages were calculated by dividing the number of faculty or student responses by the total number of faculty or students, respectively; therefore, the summed percentages may not equal 100%.

Gender-based analyses revealed significant differences in perceptions of AI use for research. Male respondents reported higher agreement than female respondents for both student use (mean ± SD: 4.02 ± 0.16 vs. 3.72 ± 0.15; *p* < 0.05) and faculty use (4.15 ± 0.14 vs. 3.89 ± 0.13; *p* < 0.05). This corresponded to a greater proportion of “strongly agree” responses among males (21.4%–28.6%) compared with females (14.6%–18.3%), while neutral responses were more common among females.

Overall, AI use was most acceptable in research and educational contexts and least acceptable in clinical settings, with generally consistent perceptions across roles but significantly stronger endorsement among male respondents for research-related applications.

### 3.4 Perceived need for further AI training

Survey responses revealed a strong perceived need for additional training or education in the use of LLM-based AI tools across all groups; yet significant differences emerged between faculty and students. 47.57% of faculty respondents strongly agreed that they required further AI training, compared to only 23.38% of students (residents, dental and hygiene students combined). When “agree” and “strongly agree” responses were combined, the majority of both groups endorsed a need for additional education, with faculty significantly more likely to express the highest level of need (*p* < 0.05). There were no statistically significant differences in perceived training needs by gender.

### 3.5 User experience ratings of LLM-based AI tools

User experience outcomes were assessed on a 5-point Likert scale, consistently indicated a positive overall perception (Table 3). Group differences were assessed using the Kruskal–Wallis rank-sum test; where the omnibus test was significant, the Tukey–Kramer–Nemenyi test was used for pairwise comparisons. When comparing user experience outcomes, only ChatGPT had a significant difference between groups. However, significant differences emerged when comparing academic roles and gender. Students reported a more favorable experience across all outcome categories compared to faculty (all *p* < 0.05). Specifically, students rated overall user experience (4.35 ± 0.21 vs. 3.77 ± 0.21, *p* < 0.01), quality of results (4.03 ± 0.22 vs. 3.60 ± 0.21, *p* < 0.05), ease of use (4.65 ± 0.18 vs. 4.00 ± 0.20, *p* < 0.001), price perception (4.49 ± 0.29 vs. 3.83 ± 0.22, *p* < 0.001), and ease of access (4.51 ± 0.19 vs. 3.94 ± 0.18, *p* < 0.001) significantly higher than faculty. Regarding gender, male respondents reported more favorable outcomes for quality of results (4.03 ± 0.23 vs. 3.68 ± 0.17, *p* < 0.05) and ease of access (4.43 ± 0.20 vs. 4.08 ± 0.16, *p* < 0.05) compared to female respondents. These group differences were evaluated using the Kruskal–Wallis rank-sum test, with *post-hoc* Tukey–Kramer–Nemenyi tests for pairwise comparisons. Use of Bard, Bing Chat, Claude, Perplexity, and Jasper was infrequent; indicating average or below-average experiences and underscoring a clear preference for ChatGPT among respondents.

**Table 3:** User-experience outcomes.

	Academic role			Gender		
Outcome category	Faculty (mean ± SD)	Student (mean ± SD)	*p*-value	Female (mean ± SD)	Male (mean ± SD)	*p*-value
Overall user experience	3.77 ± 0.21	4.35 ± 0.21	**<0.01** **	3.97 ± 0.18	4.16 ± 0.23	0.27
Quality of results	3.60 ± 0.21	4.03 ± 0.22	**<0** **.** **05** *	3.68 ± 0.17	4.03 ± 0.23	**<0** **.** **05** *
Ease of use	4.00 ± 0.20	4.65 ± 0.18	**<0** **.** **001** **	4.21 ± 0.18	4.41 ± 0.21	0.21
Price perception	3.83 ± 0.22	4.49 ± 0.29	**<0** **.** **001** **	4.10 ± 022	4.24 ± 0.30	0.37
Ease of access	3.94 ± 0.18	4.51 ± 0.19	**<0** **.** **001** **	4.08 ± 0.16	4.43 ± 0.20	**<0** **.** **05** *

The mean ratings on a 5-point Likert scale (1 = strongly disagree, 5 = strongly agree). Mean numbers were 4 across all groups and outcomes, indicating consistently positive user experience. The students reported better experiences compared to faculty in all outcome categories (all *p* < 0.05*). In quality of results and ease of access, male respondents had more favorable results than females (both *p* < 0.05*).

Bold values indicate statistically significant differences (*p* < 0.05).

*p < 0.05; **p < 0.01.

### 3.6 Qualitative insights

Open-ended responses provided additional context to the quantitative findings. Inductive thematic analysis identified three key concerns regarding AI use in dental education.

1. Over-reliance and erosion of foundational skills. Participants emphasized that AI should be an adjunct rather than a substitute for independent reasoning and learning, with concerns about reduced critical thinking. As one respondent noted, “Because this might prevent critical thinking,” while another stated, “AI should be used as a tool, not be the driver of the result.”
2. Risks of independent learning and clinical competence. Respondents cautioned that substituting AI for foundational training may hinder skill development and clinical judgment. One participant stated, “AI should never be a replacement tool for learning but it should absolutely be used to help answer questions as we are studying the material,” while another emphasized, “AI should not replace a student's/faculty's clinical judgement and problem-solving based on critical thinking.”
3. Concerns regarding reliability of AI-generated information. Participants also expressed concerns about accuracy and trustworthiness of AI outputs. One respondent noted, “AI still makes many mistakes… and may give students wrong information,” while another stated, “AI currently is an adjunctive tool and not to be used as one's only source of information. It still can provide inaccurate information.”

#### 3.6.1 Reasons for not allowing students to use AI for certain tasks

Fifty-one respondents provided written comments explaining why students should not be permitted to use AI for specific tasks. The most frequently cited reasons, across both students and faculty roles, concerned the potential negative impact on developing critical thinking, independent problem-solving, and foundational skills. Many respondents emphasized that students should first master the “basics” or “fundamentals” before incorporating AI. Concerns about overreliance were common, with several noting that students might “depend on AI and not learn” or use it as a “crutch.” Additional responses referenced the risk of inaccurate or unreliable AI-generated information, especially in clinical contexts, and the need for the ability to independently verify AI outputs. Others noted that certain tasks, such as writing assignments, examinations, and diagnostic activities, should be completed without AI's assistance to maintain academic integrity and skill acquisition.

#### 3.6.2 Reasons for not allowing faculty to use AI for certain tasks

Twenty-eight respondents elaborated on why faculty should not be permitted to use AI for certain tasks. Several respondents emphasized that faculty should demonstrate and model competence without reliance on AI, particularly for clinical teaching, to set a strong example for students. Concern was expressed that AI use for instructional or grading tasks might reduce direct engagement with learners, diminish originality in feedback, or compromise the accuracy of content delivered. Some noted that if certain AI uses were restricted for students, similar restrictions should apply to faculty. Comments also referenced a need for information validity checks, with the view that faculty should assume responsibility for confirming the accuracy of any AI-assisted material.

#### 3.6.3 Additional open-ended survey comments

In the final general comments section of the survey, 64 respondents provided additional remarks not tied to a specific question. Several reiterated points made earlier in the survey, with the most common themes including the need for clear institutional guidelines for AI use, explicit definitions of permissible and prohibited applications, and the importance of ongoing training for both students and faculty. A smaller subset emphasized the potential of AI to streamline workflows and enhance learning efficiency, while others cautioned against overreliance and underscored the necessity of verifying AI-generated information for accuracy. Multiple respondents indicated uncertainty about current institutional policies or sought more transparent communication from leadership regarding AI expectations. Some comments expressed enthusiasm for AI as an inevitable part of future dental education and practice, while others stressed that its use should always be coupled with appropriate faculty oversight and critical thinking. A few respondents also requested more opportunities to trial a variety of LLM-based AI platforms beyond ChatGPT and Grammarly AI. Analysis of these open-ended responses revealed no strong or consistent thematic differences by participant role or gender; faculty and students, as well as male and female respondents, offered similar remarks, with perspectives on AI implementation, policy, and related concerns broadly shared across all subgroups.

## 4 Discussion

This study provides a contemporary snapshot of LLM-based AI adoption, experiences, and attitudes among faculty, residents, and dental and dental hygiene students within a U.S. dental school. A clear gradient emerged in perceived appropriateness of AI use, with respondents most comfortable using AI for educational tasks, less so for research activities, and least for clinical decision-making. Consistent with prior studies in dental and health professions education, AI tools were most frequently used for academic writing and learning support, while clinical applications remained limited due to concerns regarding reliability, patient safety, and the need for independent clinical judgment (1, 2, 7). These findings align with earlier survey-based research demonstrating that students are generally more receptive to AI integration than faculty, particularly in educational contexts, whereas faculty exhibit greater caution and emphasize the need for structured training and oversight (12–15).

The prominence of LLM-based AI tools for educational tasks observed in this study aligns with prior work highlighting their instructional potential in dental education. AI chatbots have been shown to function effectively as virtual patients within constructivist learning frameworks, supporting student engagement and clinical reasoning in classroom settings (6, 11, 16–19). However, clinically oriented evaluations have demonstrated important limitations, with variability between AI-generated outputs and expert judgment in diagnostic contexts, underscoring concerns related to accuracy and contextual nuance (20). Consistent with broader professional commentary, AI adoption remains most accepted for educational purposes and approached more cautiously in clinical applications due to concerns regarding reliability, accountability, and patient safety (17–19).

The limited use of LLM-based AI tools in clinical and research contexts observed in this study is consistent with emerging evidence that, while AI is widely accepted for administrative and educational purposes, its role in clinical decision-making remains cautiously approached (2, 11, 12). Prior studies similarly report that both students and clinicians view AI as a supplementary tool rather than a replacement for professional expertise, particularly in high-stakes environments such as patient care. Qualitative findings from this study reinforce this perspective, with respondents emphasizing the importance of maintaining foundational knowledge, critical thinking, and independent diagnostic reasoning, and positioning AI as an adjunct rather than a substitute for clinical judgment.

Recent literature on next-generation educational chatbots emphasizes that, while LLMs may enhance accessibility and learning efficiency, insufficient educational guidance and excessive reliance on AI systems may contribute to passive learning behaviors and reduced critical engagement among students (21–23). The qualitative findings in the present study closely reflect these concerns, as participants repeatedly emphasized the importance of preserving learner autonomy, analytical reasoning, and active engagement when using AI-assisted tools. These findings support the need for educational strategies and institutional approaches that promote critical thinking and active cognitive engagement rather than simple task automation.

This study contributes to the growing body of literature by integrating both quantitative and qualitative data to examine not only perceptions but also actual usage patterns and training needs within a single institutional context. While prior research has often focused primarily on awareness or attitudes, the current findings provide a more comprehensive understanding of how AI is currently being used in dental education. The strong demand for additional training echoes recommendations from recent studies calling for structured curricula, ethical guidelines, and institutional policies to guide responsible AI adoption (8, 9, 11). In addition, the predominance of tools such as ChatGPT and Grammarly AI in writing and educational tasks reflects broader trends in accessibility and usability reported in the literature.

From a healthcare perspective, these findings are also consistent with human-centered AI frameworks such as the HiCARE framework, which emphasizes balancing technological innovation with ethical oversight, transparency, and responsible use in healthcare settings (24). The cautious acceptance of AI for educational purposes, alongside hesitation regarding clinical decision-making, reflects the importance of maintaining human supervision and professional accountability in healthcare education settings.

Notable strengths of this research include its inclusive sample, incorporation of both faculty and learner perspectives, and the use of mixed quantitative and qualitative methods to capture a more nuanced understanding of AI adoption. However, several limitations should be considered. First, the study was conducted at a single institution (UTSDH), which may limit generalizability to other educational settings. Although the sample included faculty, residents, and students, these groups were combined into broader analytic categories to facilitate comparison, which may obscure important subgroup differences. Second, voluntary participation introduces the potential for selection bias, and the reliance on self-reported data may be subject to recall and social desirability biases. Additionally, the sample distribution was weighted toward certain trainee groups, which may affect representativeness. Finally, as a cross-sectional study, the findings reflect a specific point in time and may evolve as AI technologies and institutional policies continue to develop.

## 5 Conclusion

Dental education appears to be at an early but rapidly evolving phase of AI integration. Consistent with the broader literature, participants expressed enthusiasm for AI's potential to enhance learning efficiency and academic productivity while also identifying concerns regarding overreliance, accuracy, and ethical use. Students demonstrated greater openness toward AI adoption, whereas faculty emphasized the need for structured training and guidance. Although clinical and research applications remain limited, AI shows considerable potential as a supplementary educational tool.

Several practical considerations may support responsible AI integration in dental education, including incorporation of AI literacy and ethics training into curricula, development of institutional guidelines regarding acceptable AI use, emphasis on critical appraisal and reflective learning when interpreting AI-generated outputs, and continued faculty oversight to ensure that AI supplements rather than replaces foundational knowledge and clinical reasoning skills.

Addressing these challenges will require a balanced, evidence-based approach integrating curriculum development, faculty training, and clear institutional policies. As conversational AI technologies continue to evolve, the findings of this study may also provide insight for other healthcare education disciplines, navigating similar opportunities and challenges associated with responsible AI integration.

## Data Availability

The original contributions presented in the study are included in the article/Supplementary Material, further inquiries can be directed to the corresponding author.
