# Supplementary material for: Evaluating artificial intelligence large language models in dental education: a cross-sectional survey on usage, perceptions, and integration at a U.S. dental school
Source: Front Digit Health. 2026 Jun 8;8:1786363. doi: 10.3389/fdgth.2026.1786363 (PMC13284074; doi:10.3389/fdgth.2026.1786363)
Supplement: Supplementary file 1 [file Datasheet1.pdf]

ChatGPT Project - Survey Questions

Start of Block: Demographics / Initial Questions

Q1 What is your current primary role?

- ○ Faculty (1)
- ○ Dental Student (2)
- ○ Dental Hygiene Student (including MSDH) (3)
- ○ Resident (4)

Q2 What year of the DDS program are you currently in?

- ○ 1st Year (1)
- ○ 2nd Year (2)
- ○ 3rd Year (3)
- ○ 4th Year (4)

Q3 What year of the DH program are you currently in?

- ○ 1st Year (1)
- ○ 2nd Year (2)
- ○ Masters of Dental Hygiene Program (3)

Q4 What residency are you undertaking?

- AEGD (1)
- Endodontics (2)
- GPR (3)
- Oral and Maxillofacial Pathology (9)
- Oral and Maxillofacial Surgery (4)
- Orthodontics (5)
- Pediatrics (6)
- Periodontics (7)
- Prosthodontics (8)

Q5 To which department are you primarily assigned?

- Diagnostic & Biomedical Science (1)
- Endodontics (2)
- General Practice and Dental Public Health (3)
- Oral & Maxillofacial Surgery (4)
- Orthodontics (5)
- Pediatric Dentistry (6)
- Periodontics & Dental Hygiene (7)
- Restorative Dentistry & Prosthodontics (8)

Q6 What is/are your role(s) in your department?

(Please select all that apply)

- ▢ Didactic Teaching (1)
- ▢ Preclinical Teaching (2)
- ▢ Clinical Teaching (3)
- ▢ Research (4)
- ▢ Administrative (5)
- ▢ Faculty Practice (6)
- ▢ Private Practice (7)

Q7 Please specify your gender:

- ○ Female (1)
- ○ Male (2)
- ○ Non-binary / third gender (3)
- ○ Prefer not to say (4)
- ○ Prefer to self-define (5) ____

Q8 What is your age (in years)?

____

Q9 To what level do you agree or disagree with the following statements?

I feel that using AI tools in the provision of dental care is:

	Strongly Disagree (10)	Disagree (11)	Neither Agree nor Disagree (12)	Agree (13)	Strongly Agree (14)
Ethical (1)	○	○	○	○	○
Cheating (2)	○	○	○	○	○
Beneficial (3)	○	○	○	○	○
Efficient (4)	○	○	○	○	○
Other (Please Specify) (5)	○	○	○	○	○

Q10 I feel that I need further training/education in the use of AI tools?

- ○ Strongly Disagree (15)
- ○ Disagree (16)
- ○ Neither Agree nor Disagree (17)
- ○ Agree (18)
- ○ Strongly Agree (19)

Q11 Your school should allow **students** to use AI for...

	Strongly Disagree (1)	Disagree (2)	Neither Agree nor Disagree (4)	Agree (5)	Strongly Agree (6)

Educational Tasks (1)	○	○	○	○	○
Clinical Tasks (2)	○	○	○	○	○
Research Tasks (3)	○	○	○	○	○

Q12 Why do you think the school should not allow **students** to use AI for certain tasks?

____

____

____

____

____

Q13 Your school should allow **faculty** to use AI for...

	Strongly Disagree (1)	Disagree (2)	Neither Agree not Disagree (4)	Agree (5)	Strongly Agree (6)
Educational Tasks (1)	○	○	○	○	○
Clinical Tasks (2)	○	○	○	○	○
Research Tasks (3)	○	○	○	○	○

Q14 Why do you think the school should not allow **faculty** to use AI for certain tasks?

____

____

____

____

____

Q15 How often have you used the following AI tools for **school-related work**?

	Have not heard of it (0)	Never (1)	Rarely (2)	Sometimes (3)	Often (4)	Always (5)
Bard (1)	o	o	o	o	o	o
Bing Chat (2)	o	o	o	o	o	o
ChatGPT (3)	o	o	o	o	o	o
Claude (4)	o	o	o	o	o	o
Grammarly AI (5)	o	o	o	o	o	o
Jasper (6)	o	o	o	o	o	o
Perplexity (7)	o	o	o	o	o	o
Other (Please Specify) (8)	o	o	o	o	o	o

End of Block: Demographics / Initial Questions

Start of Block: Bard

Q16 Considering your use of **Bard**, please rate your experience in the following categories

	Very Poor (1)	Poor (2)	Fair (3)	Good (4)	Excellent (5)
Overall user experience (1)	○	○	○	○	○
Quality of the results (2)	○	○	○	○	○
Ease of use (3)	○	○	○	○	○
Price (4)	○	○	○	○	○
Features (5)	○	○	○	○	○
Ease of access (6)	○	○	○	○	○

Q17 What specific educational / administrative tasks have you used **Bard** for?

(Please select all that apply)

- ▢ I do not use **Bard** for educational / administrative tasks (11)
- ▢ Answering clinical case-related questions (1)
- ▢ Answering quizzes/multiple choice questions (2)
- ▢ Assisting with treatment planning (3)
- ▢ Design of presentations (4)
- ▢ Understanding medical/dental concepts (5)
- ▢ Summarizing articles (6)

- [ ] Writing communications (emails,texts, etc..) (7)
- [ ] Writing essay responses (8)
- [ ] Writing for work (reports, CV, personal statements, etc.) (10)
- [ ] Other (Please specify) (9) ____

Q18 What specific educational / administrative tasks have you used **Bard** for?

(Please select all that apply)

- [ ] I do not use **Bard** for educational / administrative tasks (13)
- [ ] Assisting with treatment planning (1)
- [ ] Course / Curriculum Design (syllabi, objectives, outcomes etc.) (11)
- [ ] Creating clinical cases (2)
- [ ] Creating essay questions (3)
- [ ] Creating quizzes/multiple choice questions (4)
- [ ] Design of presentations (5)
- [ ] Grading student work (6)
- [ ] Summarizing articles (8)
- [ ] Understanding medical/dental concepts (7)
- [ ] Writing communications (emails, texts, etc.) (9)
- [ ] Writing for work (reports, CV, personal statements, etc.) (12)

- [ ] Other (Please specify) (10) ____

Q19 What specific clinical tasks have you used **Bard** for?

(Please select all that apply)

- [ ] I do not use **Bard** for clinical tasks (10)
- [ ] Diagnosis (1)
- [ ] Treatment Planning (2)
- [ ] Radiographic Interpretation (3)
- [ ] Patient Communication (4)
- [ ] Language Interpretation (5)
- [ ] Step-by-step Procedure Instructions (6)
- [ ] Pharmacological Contraindications / Adverse Reactions (7)
- [ ] Progress Notes (9)
- [ ] Other (Please Specify) (8) ____

Q20 What specific research-related tasks have you used **Bard** for?

(Please select all that apply)

- [ ] I do not use **Bard** for research-related tasks (7)
- [ ] Finding references (4)
- [ ] Performing a literature review (3)
- [ ] Reviewing articles as reviewer (8)

- ☐ Statistical analysis / Analyzing data (5)
- ☐ Summarizing articles / literature (9)
- ☐ Writing articles / manuscripts (2)
- ☐ Writing grant applications (1)
- ☐ Other (Please Specify) (6) ____

End of Block: Bard

Start of Block: Bing Chat

Q21 Considering your use of **Bing Chat**, please rate your experience in the following categories

	Very Poor (1)	Poor (2)	Fair (3)	Good (4)	Excellent (5)
Overall user experience (1)	○	○	○	○	○
Quality of the results (2)	○	○	○	○	○
Ease of use (3)	○	○	○	○	○
Price (4)	○	○	○	○	○
Features (5)	○	○	○	○	○
Ease of access (6)	○	○	○	○	○

Q22 What specific educational / administrative tasks have you used **Bing Chat** for?

(Please select all that apply)

- ☐ I do not use **Bing Chat** for educational / administrative tasks (11)
- ☐ Answering clinical case-related questions (1)
- ☐ Answering quizzes/multiple choice questions (2)
- ☐ Assisting with treatment planning (3)
- ☐ Design of presentations (4)
- ☐ Understanding medical/dental concepts (5)
- ☐ Summarizing articles (6)
- ☐ Writing communications (emails,texts, etc..) (7)
- ☐ Writing essay responses (8)
- ☐ Writing for work (reports, CV, personal statements, etc.) (10)
- ☐ Other (Please specify) (9) ____

Q23 What specific educational / administrative tasks have you used **Bing Chat** for?

(Please select all that apply)

- ☐ I do not use **Bing Chat** for educational / administrative tasks (13)
- ☐ Assisting with treatment planning (1)
- ☐ Course / Curriculum Design (syllabi, objectives, outcomes etc.) (11)
- ☐ Creating clinical cases (2)
- ☐ Creating essay questions (3)

- ☐ Creating quizzes/multiple choice questions (4)
- ☐ Design of presentations (5)
- ☐ Grading student work (6)
- ☐ Summarizing articles (8)
- ☐ Understanding medical/dental concepts (7)
- ☐ Writing communications (emails, texts, etc.) (9)
- ☐ Writing for work (reports, CV, personal statements, etc.) (12)
- ☐ Other (Please specify) (10) ____

Q24 What specific clinical tasks have you used **Bing Chat** for?

(Please select all that apply)

- ☐ I do not use **Bing Chat** for clinical tasks (10)
- ☐ Diagnosis (1)
- ☐ Treatment Planning (2)
- ☐ Radiographic Interpretation (3)
- ☐ Patient Communication (4)
- ☐ Language Interpretation (5)
- ☐ Step-by-step Procedure Instructions (6)
- ☐ Pharmacological Contraindications / Adverse Reactions (7)
- ☐ Progress Notes (9)

- [ ] Other (Please Specify) (8) ____

Q25 What specific research-related tasks have you used **Bing Chat** for?

(Please select all that apply)

- [ ] I do not use **Bing Chart** for research-related tasks (7)
- [ ] Finding references (4)
- [ ] Performing a literature review (3)
- [ ] Reviewing articles as reviewer (8)
- [ ] Statistical analysis / Analyzing data (5)
- [ ] Summarizing articles / literature (9)
- [ ] Writing articles / manuscripts (2)
- [ ] Writing grant applications (1)
- [ ] Other (Please Specify) (6) ____

End of Block: Bing Chat

Start of Block: ChatGPT

Q26 Considering your use of **ChatGPT**, please rate your experience in the following categories

	Very Poor (1)	Poor (2)	Fair (3)	Good (4)	Excellent (5)
Overall user experience (1)	○	○	○	○	○
Quality of the results (2)	○	○	○	○	○
Ease of use (3)	○	○	○	○	○

Price (4)	○	○	○	○	○
Features (5)	○	○	○	○	○
Ease of access (6)	○	○	○	○	○

Q27 What specific educational / administrative tasks have you used **ChatGPT** for?

(Please select all that apply)

- ▢ I do not use **ChatGPT** for educational / administrative tasks (11)
- ▢ Answering clinical case-related questions (1)
- ▢ Answering quizzes/multiple choice questions (2)
- ▢ Assisting with treatment planning (3)
- ▢ Design of presentations (4)
- ▢ Understanding medical/dental concepts (5)
- ▢ Summarizing articles (6)
- ▢ Writing communications (emails,texts, etc..) (7)
- ▢ Writing essay responses (8)
- ▢ Writing for work (reports, CV, personal statements, etc.) (10)
- ▢ Other (Please specify) (9) ____

Q28 What specific educational / administrative tasks have you used **ChatGPT** for?

(Please select all that apply)

- ☐ I do not use **ChatGPT** for educational / administrative tasks (13)
- ☐ Assisting with treatment planning (1)
- ☐ Course / Curriculum Design (syllabi, objectives, outcomes etc.) (11)
- ☐ Creating clinical cases (2)
- ☐ Creating essay questions (3)
- ☐ Creating quizzes/multiple choice questions (4)
- ☐ Design of presentations (5)
- ☐ Grading student work (6)
- ☐ Summarizing articles (8)
- ☐ Understanding medical/dental concepts (7)
- ☐ Writing communications (emails, texts, etc.) (9)
- ☐ Writing for work (reports, CV, personal statements, etc.) (12)
- ☐ Other (Please specify) (10) ____

Q29 What specific clinical tasks have you used **ChatGPT** for?

(Please select all that apply)

- ☐ I do not use **ChatGPT** for clinical tasks (10)
- ☐ Diagnosis (1)

- ☐ Treatment Planning (2)
- ☐ Radiographic Interpretation (3)
- ☐ Patient Communication (4)
- ☐ Language Interpretation (5)
- ☐ Step-by-step Procedure Instructions (6)
- ☐ Pharmacological Contraindications / Adverse Reactions (7)
- ☐ Progress Notes (9)
- ☐ Other (Please Specify) (8) ____

Q30 What specific research-related tasks have you used **ChatGPT** for?

(Please select all that apply)

- ☐ I do not use **ChatGPT** for research-related tasks (7)
- ☐ Finding references (4)
- ☐ Performing a literature review (3)
- ☐ Reviewing articles as reviewer (8)
- ☐ Statistical analysis / Analyzing data (5)
- ☐ Summarizing articles / literature (9)
- ☐ Writing articles / manuscripts (2)
- ☐ Writing grant applications (1)
- ☐ Other (Please Specify) (6) ____

End of Block: ChatGPT

Start of Block: Claude

Q31 Considering your use of **Claude**, please rate your experience in the following categories

	Very Poor (1)	Poor (2)	Fair (3)	Good (4)	Excellent (5)
Overall user experience (1)	○	○	○	○	○
Quality of the results (2)	○	○	○	○	○
Ease of use (3)	○	○	○	○	○
Price (4)	○	○	○	○	○
Features (5)	○	○	○	○	○
Ease of access (6)	○	○	○	○	○

Q32 What specific educational / administrative tasks have you used **Claude** for?

(Please select all that apply)

▢ I do not use **Claude** for educational / administrative tasks (11)

▢ Answering clinical case-related questions (1)

▢ Answering quizzes/multiple choice questions (2)

▢ Assisting with treatment planning (3)

▢ Design of presentations (4)

- ☐ Understanding medical/dental concepts (5)
- ☐ Summarizing articles (6)
- ☐ Writing communications (emails,texts, etc..) (7)
- ☐ Writing essay responses (8)
- ☐ Writing for work (reports, CV, personal statements, etc.) (10)
- ☐ Other (Please specify) (9) ____

Q33 What specific educational / administrative tasks have you used **Claude** for?

(Please select all that apply)

- ☐ I do not use **Claude** for educational / administrative tasks (13)
- ☐ Assisting with treatment planning (1)
- ☐ Course / Curriculum Design (syllabi, objectives, outcomes etc.) (11)
- ☐ Creating clinical cases (2)
- ☐ Creating essay questions (3)
- ☐ Creating quizzes/multiple choice questions (4)
- ☐ Design of presentations (5)
- ☐ Grading student work (6)
- ☐ Summarizing articles (8)
- ☐ Understanding medical/dental concepts (7)

- Writing communications (emails, texts, etc.) (9)
- Writing for work (reports, CV, personal statements, etc.) (12)
- Other (Please specify) (10) ____

Q34 What specific clinical tasks have you used **Claude** for?

(Please select all that apply)

- I do not use **Claude** for clinical tasks (10)
- Diagnosis (1)
- Treatment Planning (2)
- Radiographic Interpretation (3)
- Patient Communication (4)
- Language Interpretation (5)
- Step-by-step Procedure Instructions (6)
- Pharmacological Contraindications / Adverse Reactions (7)
- Progress Notes (9)
- Other (Please Specify) (8) ____

Q35 What specific research-related tasks have you used **Claude** for?

(Please select all that apply)

- I do not use **Claude** for research-related tasks (7)
- Finding references (4)

- ☐ Performing a literature review (3)
- ☐ Reviewing articles as reviewer (8)
- ☐ Statistical analysis / Analyzing data (5)
- ☐ Summarizing articles / literature (9)
- ☐ Writing articles / manuscripts (2)
- ☐ Writing grant applications (1)
- ☐ Other (Please Specify) (6) ____

End of Block: Claude

Start of Block: Grammarly AI

Q36 Considering your use of **Grammarly AI**, please rate your experience in the following categories

	Very Poor (1)	Poor (2)	Fair (3)	Good (4)	Excellent (5)
Overall user experience (1)	○	○	○	○	○
Quality of the results (2)	○	○	○	○	○
Ease of use (3)	○	○	○	○	○
Price (4)	○	○	○	○	○
Features (5)	○	○	○	○	○
Ease of access (6)	○	○	○	○	○

Q37 What specific educational / administrative tasks have you used **Grammarly AI** for?

(Please select all that apply)

- ▢ I do not use **Grammarly AI** for educational / administrative tasks (11)
- ▢ Answering clinical case-related questions (1)
- ▢ Answering quizzes/multiple choice questions (2)
- ▢ Assisting with treatment planning (3)
- ▢ Design of presentations (4)
- ▢ Understanding medical/dental concepts (5)
- ▢ Summarizing articles (6)
- ▢ Writing communications (emails,texts, etc..) (7)
- ▢ Writing essay responses (8)
- ▢ Writing for work (reports, CV, personal statements, etc.) (10)
- ▢ Other (Please specify) (9) ____

Q38 What specific educational / administrative tasks have you used **Grammarly AI** for?

(Please select all that apply)

- ▢ I do not use **Grammarly AI** for educational / administrative tasks (13)
- ▢ Assisting with treatment planning (1)
- ▢ Course / Curriculum Design (syllabi, objectives, outcomes etc.) (11)

- ☐ Creating clinical cases (2)
- ☐ Creating essay questions (3)
- ☐ Creating quizzes/multiple choice questions (4)
- ☐ Design of presentations (5)
- ☐ Grading student work (6)
- ☐ Summarizing articles (8)
- ☐ Understanding medical/dental concepts (7)
- ☐ Writing communications (emails, texts, etc.) (9)
- ☐ Writing for work (reports, CV, personal statements, etc.) (12)
- ☐ Other (Please specify) (10) ____

Q39 What specific clinical tasks have you used **Grammarly AI** for?

(Please select all that apply)

- ☐ I do not use **Grammarly AI** for clinical tasks (10)
- ☐ Diagnosis (1)
- ☐ Treatment Planning (2)
- ☐ Radiographic Interpretation (3)
- ☐ Patient Communication (4)
- ☐ Language Interpretation (5)
- ☐ Step-by-step Procedure Instructions (6)

- ▢ Pharmacological Contraindications / Adverse Reactions (7)
- ▢ Progress Notes (9)
- ▢ Other (Please Specify) (8) ____

Q40 What specific research-related tasks have you used **Grammarly AI** for?

(Please select all that apply)

- ▢ I do not use **Grammarly AI** for research-related tasks (7)
- ▢ Finding references (4)
- ▢ Performing a literature review (3)
- ▢ Reviewing articles as reviewer (8)
- ▢ Statistical analysis / Analyzing data (5)
- ▢ Summarizing articles / literature (9)
- ▢ Writing articles / manuscripts (2)
- ▢ Writing grant applications (1)
- ▢ Other (Please Specify) (6) ____

End of Block: Grammarly AI

Start of Block: Jasper

Q41 Considering your use of **Jasper**, please rate your experience in the following categories

	Very Poor (1)	Poor (2)	Fair (3)	Good (4)	Excellent (5)
Overall user experience (1)	○	○	○	○	○

Quality of the results (2)	○	○	○	○	○
Ease of use (3)	○	○	○	○	○
Price (4)	○	○	○	○	○
Features (5)	○	○	○	○	○
Ease of access (6)	○	○	○	○	○

Q42 What specific educational / administrative tasks have you used **Jasper** for?

(Please select all that apply)

- ▢ I do not use **Jasper** for educational / administrative tasks (11)
- ▢ Answering clinical case-related questions (1)
- ▢ Answering quizzes/multiple choice questions (2)
- ▢ Assisting with treatment planning (3)
- ▢ Design of presentations (4)
- ▢ Understanding medical/dental concepts (5)
- ▢ Summarizing articles (6)
- ▢ Writing communications (emails,texts, etc..) (7)
- ▢ Writing essay responses (8)

- ☐ Writing for work (reports, CV, personal statements, etc.) (10)
- ☐ Other (Please specify) (9) ____

Q43 What specific educational / administrative tasks have you used **Jasper** for?

(Please select all that apply)

- ☐ I do not use **Jasper** for educational / administrative tasks (13)
- ☐ Assisting with treatment planning (1)
- ☐ Course / Curriculum Design (syllabi, objectives, outcomes etc.) (11)
- ☐ Creating clinical cases (2)
- ☐ Creating essay questions (3)
- ☐ Creating quizzes/multiple choice questions (4)
- ☐ Design of presentations (5)
- ☐ Grading student work (6)
- ☐ Summarizing articles (8)
- ☐ Understanding medical/dental concepts (7)
- ☐ Writing communications (emails, texts, etc.) (9)
- ☐ Writing for work (reports, CV, personal statements, etc.) (12)
- ☐ Other (Please specify) (10) ____

Q44 What specific clinical tasks have you used **Jasper** for?

(Please select all that apply)

- ▢ I do not use **Jasper** for clinical tasks (10)
- ▢ Diagnosis (1)
- ▢ Treatment Planning (2)
- ▢ Radiographic Interpretation (3)
- ▢ Patient Communication (4)
- ▢ Language Interpretation (5)
- ▢ Step-by-step Procedure Instructions (6)
- ▢ Pharmacological Contraindications / Adverse Reactions (7)
- ▢ Progress Notes (9)
- ▢ Other (Please Specify) (8) ____

Q45 What specific research-related tasks have you used **Jasper** for?

(Please select all that apply)

- ▢ I do not use **Jasper** for research-related tasks (7)
- ▢ Finding references (4)
- ▢ Performing a literature review (3)
- ▢ Reviewing articles as reviewer (8)
- ▢ Statistical analysis / Analyzing data (5)
- ▢ Summarizing articles / literature (9)

- ☐ Writing articles / manuscripts (2)
- ☐ Writing grant applications (1)
- ☐ Other (Please Specify) (6) ____

End of Block: Jasper

Start of Block: Perplexity

Q46 Considering your use of **Perplexity**, please rate your experience in the following categories

	Very Poor (1)	Poor (2)	Fair (3)	Good (4)	Excellent (5)
Overall user experience (1)	○	○	○	○	○
Quality of the results (2)	○	○	○	○	○
Ease of use (3)	○	○	○	○	○
Price (4)	○	○	○	○	○
Features (5)	○	○	○	○	○
Ease of access (6)	○	○	○	○	○

Q47 What specific educational / administrative tasks have you used **Perplexity** for?

(Please select all that apply)

- ☐ I do not use **Jasper** for educational / administrative tasks (11)
- ☐ Answering clinical case-related questions (1)

- ☐ Answering quizzes/multiple choice questions (2)
- ☐ Assisting with treatment planning (3)
- ☐ Design of presentations (4)
- ☐ Understanding medical/dental concepts (5)
- ☐ Summarizing articles (6)
- ☐ Writing communications (emails,texts, etc..) (7)
- ☐ Writing essay responses (8)
- ☐ Writing for work (reports, CV, personal statements, etc.) (10)
- ☐ Other (Please specify) (9) ____

Q48 What specific educational / administrative tasks have you used **Perplexity** for?

(Please select all that apply)

- ☐ I do not use **Perplexity** for educational / administrative tasks (13)
- ☐ Assisting with treatment planning (1)
- ☐ Course / Curriculum Design (syllabi, objectives, outcomes etc.) (11)
- ☐ Creating clinical cases (2)
- ☐ Creating essay questions (3)
- ☐ Creating quizzes/multiple choice questions (4)
- ☐ Design of presentations (5)

- ☐ Grading student work (6)
- ☐ Summarizing articles (8)
- ☐ Understanding medical/dental concepts (7)
- ☐ Writing communications (emails, texts, etc.) (9)
- ☐ Writing for work (reports, CV, personal statements, etc.) (12)
- ☐ Other (Please specify) (10) ____

Q49 What specific clinical tasks have you used **Perplexity** for?

(Please select all that apply)

- ☐ I do not use **Perplexity** for clinical tasks (10)
- ☐ Diagnosis (1)
- ☐ Treatment Planning (2)
- ☐ Radiographic Interpretation (3)
- ☐ Patient Communication (4)
- ☐ Language Interpretation (5)
- ☐ Step-by-step Procedure Instructions (6)
- ☐ Pharmacological Contraindications / Adverse Reactions (7)
- ☐ Progress Notes (9)
- ☐ Other (Please Specify) (8) ____

Q50 What specific research-related tasks have you used **Perplexity** for?

(Please select all that apply)

- ▢ I do not use **Perplexity** for research-related tasks (7)
- ▢ Finding references (4)
- ▢ Performing a literature review (3)
- ▢ Reviewing articles as reviewer (8)
- ▢ Statistical analysis / Analyzing data (5)
- ▢ Summarizing articles / literature (9)
- ▢ Writing articles / manuscripts (2)
- ▢ Writing grant applications (1)
- ▢ Other (Please Specify) (6) ____

End of Block: Perplexity

Start of Block: Other

Q51 Considering your use of **${Q15/ChoiceTextEntryValue/8}**, please rate your experience in the following categories

	Very Poor (1)	Poor (2)	Fair (3)	Good (4)	Excellent (5)
Overall user experience (1)	○	○	○	○	○
Quality of the results (2)	○	○	○	○	○
Ease of use (3)	○	○	○	○	○
Price (4)	○	○	○	○	○

Features (5)	○	○	○	○	○
Ease of access (6)	○	○	○	○	○

Q52 What specific educational / administrative tasks have you used **${Q15/ChoiceTextEntryValue/8}** for?

(Please select all that apply)

- ▢ I do not use **${Q15/ChoiceTextEntryValue/8}** for educational / administrative tasks (11)
- ▢ Answering clinical case-related questions (1)
- ▢ Answering quizzes/multiple choice questions (2)
- ▢ Assisting with treatment planning (3)
- ▢ Design of presentations (4)
- ▢ Understanding medical/dental concepts (5)
- ▢ Summarizing articles (6)
- ▢ Writing communications (emails,texts, etc..) (7)
- ▢ Writing essay responses (8)
- ▢ Writing for work (reports, CV, personal statements, etc.) (10)
- ▢ Other (Please specify) (9) ____

Q53 What specific educational / administrative tasks have you used **${Q15/ChoiceTextEntryValue/8}** for?

(Please select all that apply)

- ▢ I do not use **${Q15/ChoiceTextEntryValue/8}** for educational / administrative tasks (13)
- ▢ Assisting with treatment planning (1)
- ▢ Course / Curriculum Design (syllabi, objectives, outcomes etc.) (11)
- ▢ Creating clinical cases (2)
- ▢ Creating essay questions (3)
- ▢ Creating quizzes/multiple choice questions (4)
- ▢ Design of presentations (5)
- ▢ Grading student work (6)
- ▢ Summarizing articles (8)
- ▢ Understanding medical/dental concepts (7)
- ▢ Writing communications (emails, texts, etc.) (9)
- ▢ Writing for work (reports, CV, personal statements, etc.) (12)
- ▢ Other (Please specify) (10) ____

Q54 What specific clinical tasks have you used **${Q15/ChoiceTextEntryValue/8}** for?

(Please select all that apply)

- ▢ I do not use **${Q15/ChoiceTextEntryValue/8}** for clinical tasks (10)
- ▢ Diagnosis (1)
- ▢ Treatment Planning (2)

- ☐ Radiographic Interpretation (3)
- ☐ Patient Communication (4)
- ☐ Language Interpretation (5)
- ☐ Step-by-step Procedure Instructions (6)
- ☐ Pharmacological Contraindications / Adverse Reactions (7)
- ☐ Progress Notes (9)
- ☐ Other (Please Specify) (8) ____

Q55 What specific research-related tasks have you used **${Q15/ChoiceTextEntryValue/8}** for? (Please select all that apply)

- ☐ I do not use **${Q15/ChoiceTextEntryValue/8}** for research-related tasks (7)
- ☐ Finding references (4)
- ☐ Performing a literature review (3)
- ☐ Reviewing articles as reviewer (8)
- ☐ Statistical analysis / Analyzing data (5)
- ☐ Summarizing articles / literature (9)
- ☐ Writing articles / manuscripts (2)
- ☐ Writing grant applications (1)
- ☐ Other (Please Specify) (6) ____

End of Block: Other

Start of Block: Conclusion

Q56 Please use this box to provide any feedback, comments, questions, ideas or opinions about the use of AI tools

____

____

____

____

____

End of Block: Conclusion
